# Prospective role of NSAIDs with antiviral properties for pharmacological management of postsurgical procedures during COVID-19

**DOI:** 10.1097/JS9.0000000000000101

**Published:** 2023-02-16

**Authors:** Firzan Nainu, Sukamto S. Mamada, Talha B. Emran

**Affiliations:** a Department of Pharmacy, Faculty of Pharmacy, Hasanuddin University, Makassar, Indonesia; b Department of Pharmacy, BGC Trust University Bangladesh, Chittagong; c Department of Pharmacy, Faculty of Allied Health Sciences, Daffodil International University, Dhaka, Bangladesh

*Dear Editor*,

Almost 3 years since its first case report, coronavirus disease 2019 (COVID-19) remains one of the most discussed topics in the world. Severe acute respiratory syndrome coronavirus 2 (SARS-CoV-2), the causative agent of COVID-19, infects host cells, mainly in the respiratory tracts, via immediate attachment to the angiotensin-converting enzyme type 2 receptor at the surface of host cells^1^. SARS-CoV-2-infected patients may then develop several symptoms, including fever, body pain, and malaise^2^,^3^. Some of these symptoms can also develop after patients undergo surgical procedures^4^. Indeed, during the early times of COVID-19 and even until now, surgical procedures face great challenges due to the high number of COVID-19 cases and possible viral transmission before, during, and after surgical procedures were taken^5^.

To manage fever and inflammatory-related symptoms, antipyretics, and analgesics, classified as NSAIDs, are commonly used^6^. NSAID drugs act via the inhibition of arachidonic acid conversion to prostaglandins. This mechanism of action has been suggested to play a major role in the inhibition of pain sensation and resolution of inflammation^7^. Apart from their desired pharmacological effects, some NSAIDs have been suggested to cause immunosuppression, and this has been a subject of scientific debate^8^. In this correspondence piece, we would like to raise the awareness about the immunosuppressive effect of NSAIDs, which is quite debatable, as well as their documented antiviral activity against major viruses, including SARS-CoV-2. Evidence on these two seemingly unrelated pharmacological effects shall be critically assessed and carefully managed to achieve the best use of NSAIDs in the postsurgical management amid the emerging and re-emerging of viral infectious diseases.

How actually NSAIDs exert their immunosuppressive effect? Kazama *et al.* 9 reported that immunosuppressive actions of NSAIDs are associated with their ability to inhibit the delayed rectifier K^+^-channels (Kv1.3 channels) highly expressed in the T lymphocytes, which are pivotal for facilitating lymphocytes activation and proliferation. As a result, the immune response of T lymphocytes is suppressed. Pharmacologically, this inhibitory mechanism should be considered a novel strategy to block the emergence of cytokine storms in COVID-19 patients^10^. The inhibition of lymphocyte activation might also be linked to the inhibition of p38 mitogen-activated protein kinases (MAPK)^11^,^12^. This pathway is responsible for mediating several biological processes due to the various stress stimuli (e.g. microbes with their products and cytokines). In response to these stimuli, macrophages are activated followed by the activation of signaling pathways responsible for producing inflammatory mediators.

Of several pathways involved in inflammatory cascades, the MAPK pathways play a significant role^11^. These pathways could induce nuclear factor-κB activation, which is heavily involved in the process of inflammation and immune responses, including the production of proinflammatory cytokines, such as tumor necrosis factor-alpha^11^,^13^. It has also been demonstrated that p38 MAPK activation is closely linked to the expression of the COX-2 gene and stabilization of COX-2 posttranscription^12^. It has long been noted that NSAIDs exert their anti-inflammatory action by blocking the activity of enzymes called cyclooxygenases. This enzyme is responsible for catalyzing the conversion of arachidonic acid to inflammatory mediators, including prostaglandins, followed by their binding to the appropriate receptors. This binding would eventually trigger inflammatory responses.

Several studies have reported NSAIDs’ ability to disturb the production and action of cytokines and antibodies^14^–^16^. In the current conditions, in the cases of COVID-19, the use of NSAIDs has been reported to dampen the excessive antibodies and cytokine responses^15^. This use might be helpful given that in SARS-CoV-2 infection, the expression of COX-2 shows upregulation^15^. Taken together, the use of analgesics/antipyretics/NSAIDs has been long accepted in alleviating the excessive inflammatory events which are often had by patients suffering from viral infections, including COVID-19. Furthermore, a rapid systematic review revealed that current evidence remains inadequate to support the assumption that NSAIDs have a negative impact on the adverse events, acute healthcare utilization, long-term survival, or quality of life in patients with COVID-19^17^. Hence, the use of NSAIDs in the management of inflammation and pain-related symptoms may still clinically relevant during the times of COVID-19.

One of the worldwide priorities is to ensure effective antiviral agents against emerging and re-emerging viral diseases are available^18^. To support this effort, researchers thrived to discover new antiviral candidates as well as repurpose the already available drugs. To date, several NSAIDs have been shown to yield antiviral properties, with promising effects against certain human viruses, including SARS-CoV-2 (Table 1). Although research looking at the antiviral activities of NSAIDs is still ongoing, there is a potential path to be taken for future disease management in the case of viral infection. Naproxen, for example, was demonstrated as a potent antiviral against both influenza A virus and SARS-CoV-2^23^,^25^. In addition to naproxen, one of the most commonly used NSAIDs, paracetamol, has been demonstrated to alter the expression of genes involved in the entry of SARS-CoV-2 as well as arachidonic acid metabolism^31^. Furthermore, another group of investigators have also reported the antiviral potential of indomethacin against SARS-CoV^21^ and SARS-CoV-2^32^ in the in-vitro settings, adding more prospective antiviral agents in the list. In addition to their promising effect against SARS-CoV-2, some NSAIDs have also been shown active against several human viruses, including dengue virus, chikungunya virus, and human rhinoviruses (Table 1).

**Table 1:** List of NSAIDs with prospective antiviral properties against human viruses

Drugs	Key findings	References
Antrafenine	Using in-silico analysis, antrafenine was shown to yield good antiviral activity against DENV-2 by targeting virus envelope glycoprotein	19
Aspirin	Aspirin produced no antiviral activity against DNA viruses (HSV-1 and Adeno-5). However, its antiviral effect can be seen against RNA viruses in the in-vitro setting: low activity against respiratory syncytial virus and high activity against influenza A H1N1 virus and human rhinoviruses	20
Indomethacin	Indomethacin demonstrated a great potency in reducing viral replication in both SARS-CoV-infected Vero and -infected A549 cells. Antiviral activity of indomethacin might not be mediated by cyclooxygenase blockage, viral infectivity, and blockage of either binding or entry of the virus. Instead, the antiviral effect of indomethacin might be related to its ability to inhibit viral protein synthesis	21
Mefenamic acid	In combination with ribavirin, mefenamic acid showed potent in-vitro and in-vivo antiviral activity against the chikungunya virus	22
Naproxen	Naproxen demonstrated its antiviral activity against influenza A virus (H1N1 and H3N2) in the in-vitro and in-vivo setting	23
Naproxen	In-vivo study in mice showed that naproxen halted the nuclear export of nucleoproteins of influenza A and B viruses leading to the inhibition of viral replication. This action is mediated by the ability of naproxen to directly bind to the viral nucleoprotein	24
Naproxen	Naproxen demonstrated antiviral properties against SARS-CoV-2 using in-silico (molecular docking analysis using Autodock Vina) and in-vitro (SARS-CoV-2-infected Vero E6 and HAE cells) approaches	25
Piroxicam	Piroxicam yielded a promising antiviral activity, in combination with azithromycin, in the treatment of SARS-CoV-2	26
Andrographolide, etanercept, adalimumab, infliximab, golimumab, olsalazine, myricetin, and ketoprofen	A network bioinformatics study using the AutoSeed program equipped with the AutoNet program to build the protein network showed that several analgesics, antipyretic and anti-inflammatory drugs had potencies against SARS-CoV-2	27
Lenalidomide, celecoxib, sulfasalazine, tenoxicam, meclofenamic acid, and loxoprofen	A meta-analysis approach proposed the potencies of several drugs to be further investigated as anti-SARS-CoV-2 indicated by a high negative tau score. Their antiviral activity might be related to their action on inhibiting cyclooxygenase-2 (COX-2). It has been demonstrated that COX-2 might have a role in mediating the entry of coronavirus^28^. Furthermore, another study demonstrated that COX-2 could limit the actions of antiviral cytokines. Thus, COX-2 inhibitors might play a significant role in enhancing the host antiviral immunity^29^	28–30

CC50, 50% cytotoxic concentration; COX-2, cyclooxygenase-2; DENV-2, dengue virus serotype 2; HAE cells, human airway epithelial cells; hpi, hours postinfection; HSV-1, human herpesvirus simplex virus type 1.

In conclusion, relevant evidence suggested that certain NSAIDs with their combinatorial anti-inflammatory and antiviral effects could provide more efficient results in alleviating the respiratory distress symptoms and hyperinflammatory responses caused by COVID-19. Such pharmacological approach shall bring tremendous efficacy for the patients suffering from COVID-19-related cytokine storm or similar symptoms caused by other viral diseases.

## Ethical approval

Not applicable.

## Sources of funding

None.

## Authors’ contribution

F.N. and T.B.E designed the study. F.N. and S.S.M. wrote the first draft. F.N., S.S.M., and T.B.E. updated the manuscript. F.N., S.S.M., and T.B.E. reviewed the final draft. All authors have critically reviewed and approved the final draft and are responsible for the content and similarity index of the manuscript.

## Conflicts of interest disclosure

The authors declare that they have no financial conflicts of interest with regard to the consent of this report.

## Research registration unique identifying number (UIN)

None.

## Guarantor

Firzan Nainu and Talha Bin Emran.

## Provenance and peer review

Not commissioned, internally peer-reviewed.
